# Correction: Rab10 regulates neuropeptide release by maintaining Ca^2+^ homeostasis and protein synthesis

**DOI:** 10.7554/eLife.109889

**Published:** 2025-11-14

**Authors:** Jian Dong, Miao Chen, Jan R T van Weering, Natalia Domínguez, Ka Wan Li, August B Smit, Ruud F Toonen, Matthijs Verhage

**Keywords:** Mouse

Dong J, Chen M, van Weering JRT, Domínguez N, Li KW, Smit AB, Toonen RF Verhage M. 2025. Rab10 regulates neuropeptide release by maintaining Ca^2+^ homeostasis and protein synthesis. *eLife* **13**:RP94930. doi: 10.7554/eLife.94930.

Published 2 April 2025

During a post-publication review, the authors realized that Dr. Natalia Domínguez was inadvertently omitted from the author list. Dr. Domínguez made substantial experimental contributions during the early phase of the Rab10 project including participating in the conception of the idea of the knockdown approach, the design and validation of Rab10 shRNAs, generation of Rab10 rescue constructs, and performance of initial live-cell imaging experiments that revealed reduced dense-core vesicle release upon Rab10 knockdown. These data provided an essential foundation for the subsequent work reported in the article.

Dr. Domínguez left the laboratory several years before the continuation and completion of the project, and the omission occurred due to the long-time span between the initial and final stages of the work.

In addition, the authors identified a typographical error in the published author list: Miao Chen was incorrectly listed as Mian Chen. This has now been corrected. All authors have agreed to the change.

The details of Dr. Domínguez’s contributions are given below:

Dr. Domínguez contributed to the conception of the knockdown approach, designed and validated Rab10 shRNAs, generated Rab10 rescue constructs, and performed the initial live-cell imaging experiments.

**New author list:** Jian Dong, Miao Chen, Jan RT van Weering, Natalia Domínguez**,** Ka Wan Li, August B Smit, Ruud F Toonen, Matthijs Verhage

**Original author list:** Jian Dong, Mian Chen, Jan RT van Weering, Ka Wan Li, August B Smit, Ruud F Toonen, Matthijs Verhage

**Details for the omitted author:**

Natalia Domínguez

Affiliations: Department of Functional Genomics, Center for Neurogenomics and Cognitive Research (CNCR), Vrije Universiteit (VU) Amsterdam, Amsterdam, Netherlands; Department of Clinical Genetics, Center for Neurogenomics and Cognitive Research (CNCR), University Medical Center Amsterdam, Amsterdam, Netherlands

Present address: Departamento de Bioquímica, Microbiología, Biología Celular y Genética, Universidad de La Laguna (ULL), La Laguna (Tenerife), Spain.

Contribution: Investigation; Validation; Methodology

Competing interests: No competing interests declared.

The article has been corrected accordingly.

