# Supplementary figures and images for: Rab10 regulates neuropeptide release by maintaining Ca2+ homeostasis and protein synthesis
Source: eLife. 2025 Apr 2;13:RP94930. doi: 10.7554/eLife.94930 (PMC11964448; doi:10.7554/eLife.94930)

Culture #1

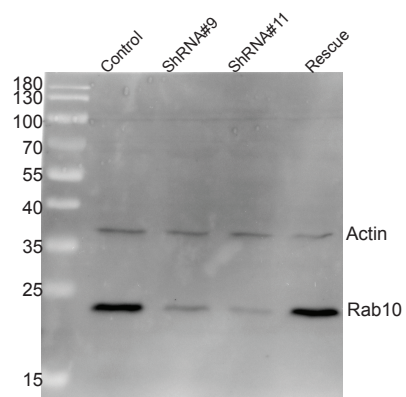

Culture #2

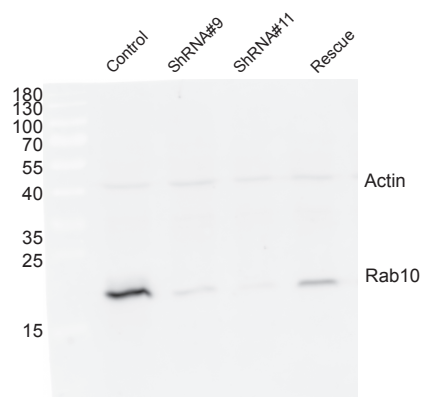

Culture #3

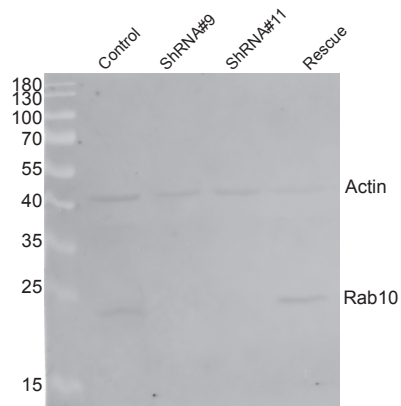

Culture #4\_Rab10

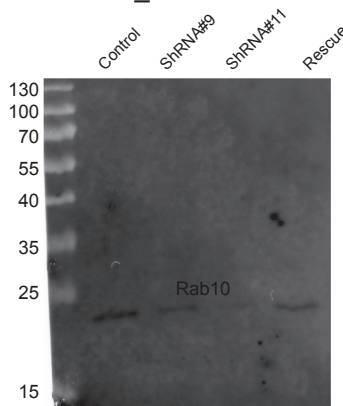

Culture #4\_Actin

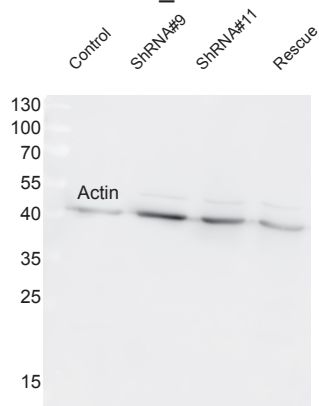

Supplement: Figure 1—source data 1. [file elife-94930-fig1-data1.zip › Figure 1_Source Data 1.pdf]

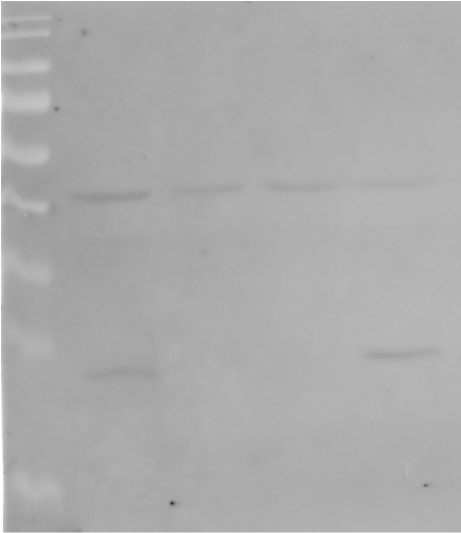

Supplement: Figure 1—source data 2. [file elife-94930-fig1-data2.zip › Figure 1_Source Data 2/w3.tif]

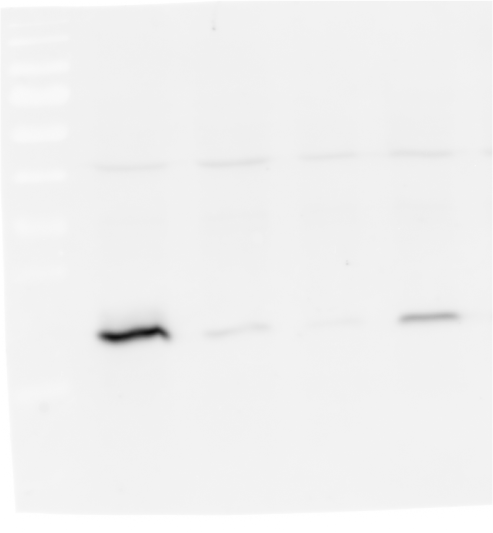

Supplement: Figure 1—source data 2. [file elife-94930-fig1-data2.zip › Figure 1_Source Data 2/w2.tif]

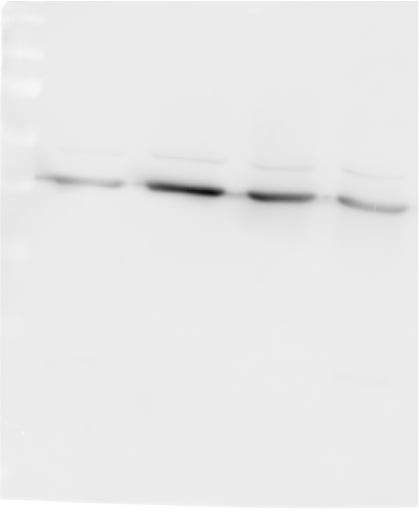

Supplement: Figure 1—source data 2. [file elife-94930-fig1-data2.zip › Figure 1_Source Data 2/w4_actin.tif]

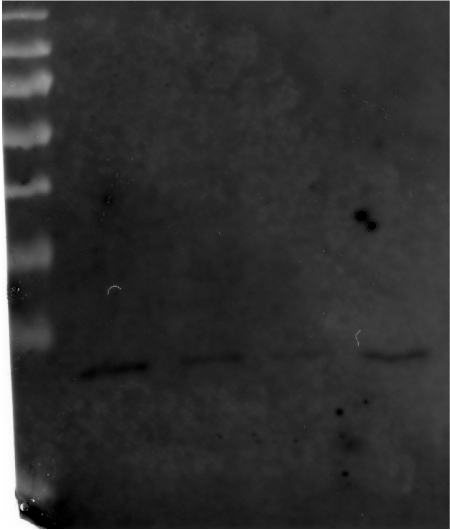

Supplement: Figure 1—source data 2. [file elife-94930-fig1-data2.zip › Figure 1_Source Data 2/w4_Rab10.tif]

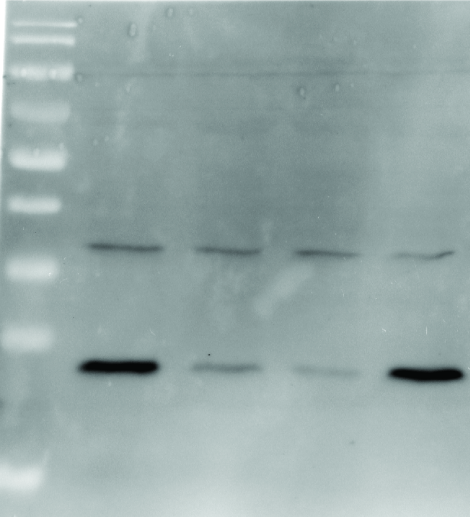

Supplement: Figure 1—source data 2. [file elife-94930-fig1-data2.zip › Figure 1_Source Data 2/w1.tif]

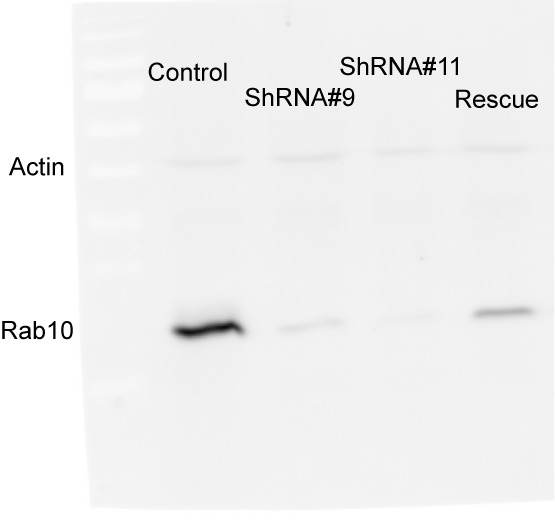

Supplement: Figure 1—source data 2. [file elife-94930-fig1-data2.zip › Figure 1_Source Data 2/w4.tif]

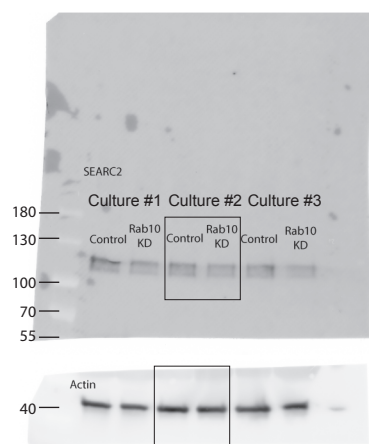

Supplement: Figure 5—source data 1. [file elife-94930-fig5-data1.zip › Figure 5A_Source Data 1.pdf]

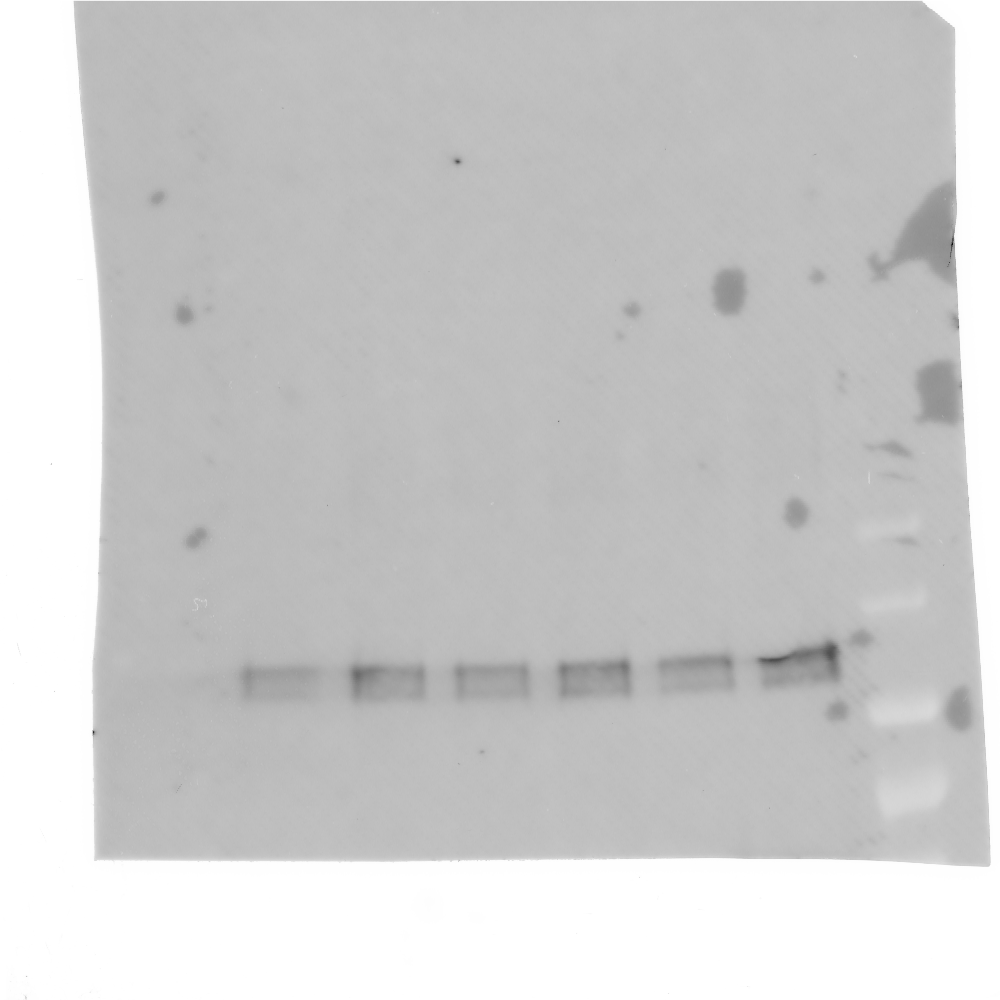

Supplement: Figure 5—source data 2. [file elife-94930-fig5-data2.zip › serca2.tif]

Culture #1

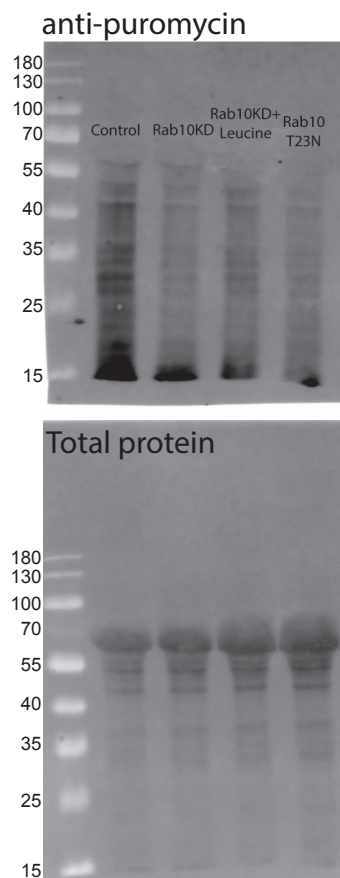

Culture #2

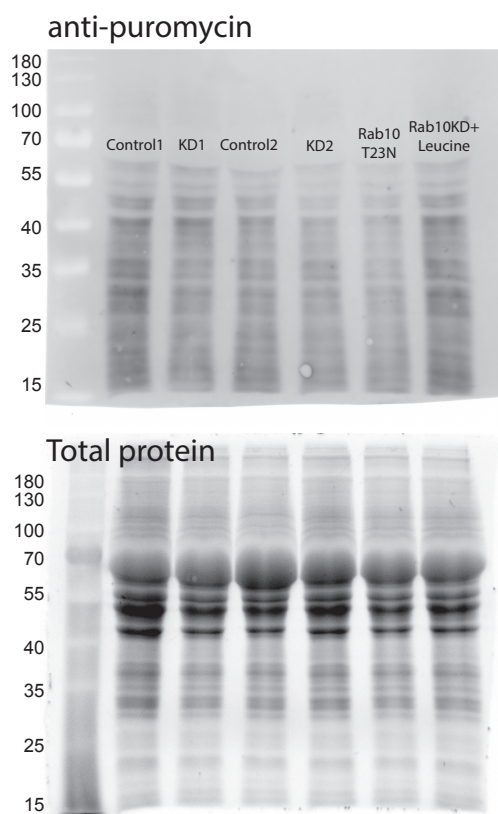

Culture #3

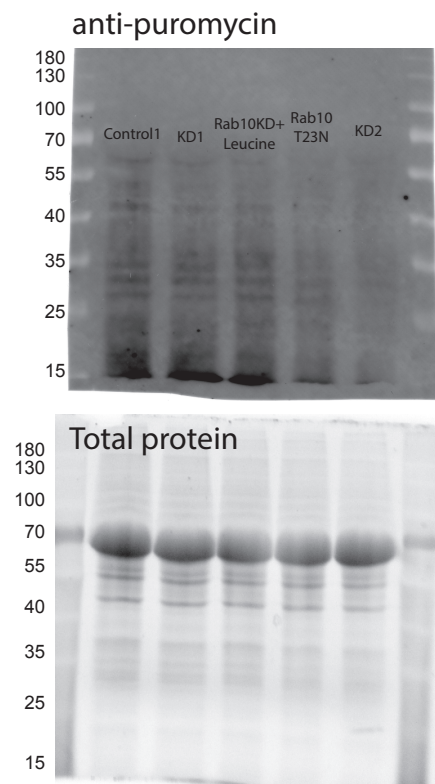

Supplement: Figure 8—source data 1. [file elife-94930-fig8-data1.zip › Figure 8A_Source Data 1.pdf]

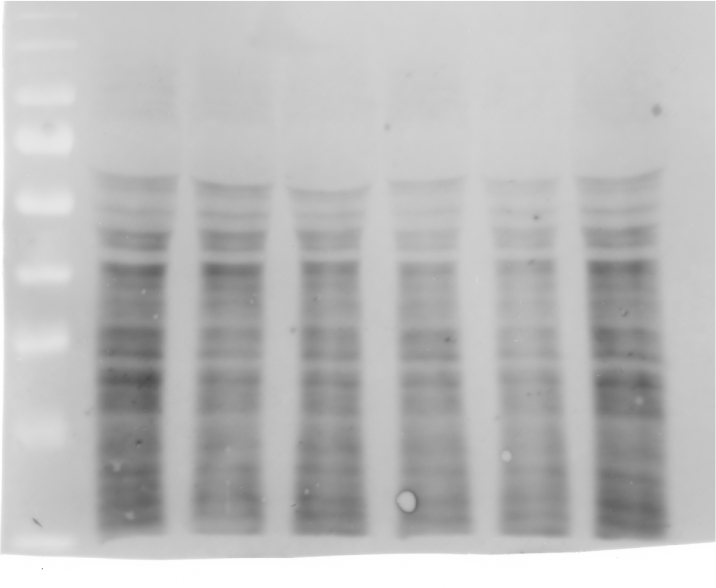

Supplement: Figure 8—source data 2. [file elife-94930-fig8-data2.zip › Figure 8A_Source Data 2/w2_puromycin.tif]

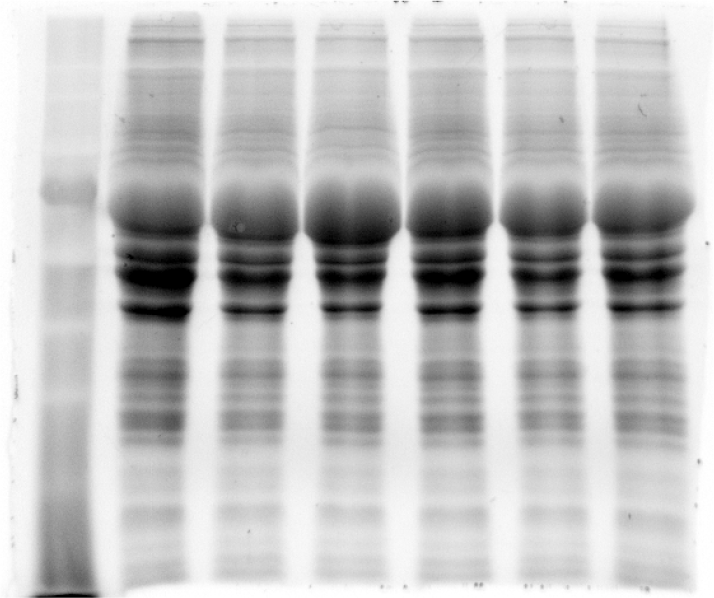

Supplement: Figure 8—source data 2. [file elife-94930-fig8-data2.zip › Figure 8A_Source Data 2/w2_total.tif]

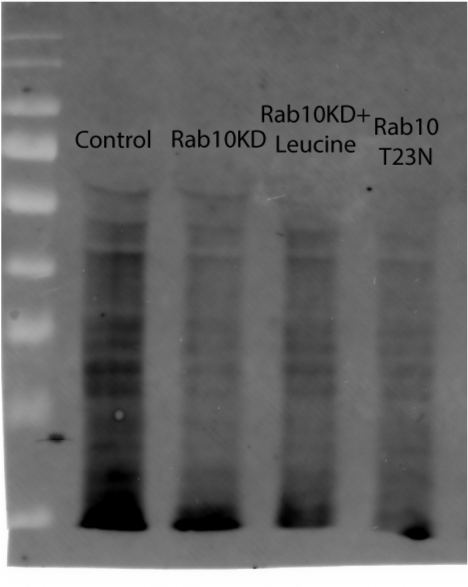

Supplement: Figure 8—source data 2. [file elife-94930-fig8-data2.zip › Figure 8A_Source Data 2/w1_puromycin.tif]

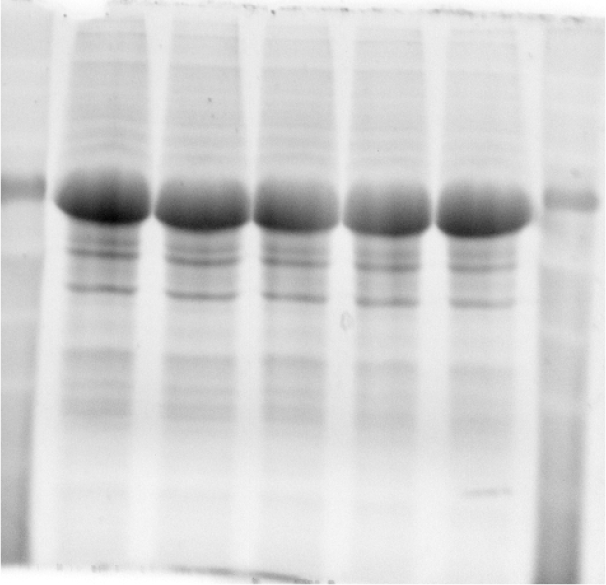

Supplement: Figure 8—source data 2. [file elife-94930-fig8-data2.zip › Figure 8A_Source Data 2/w3_total.tif]

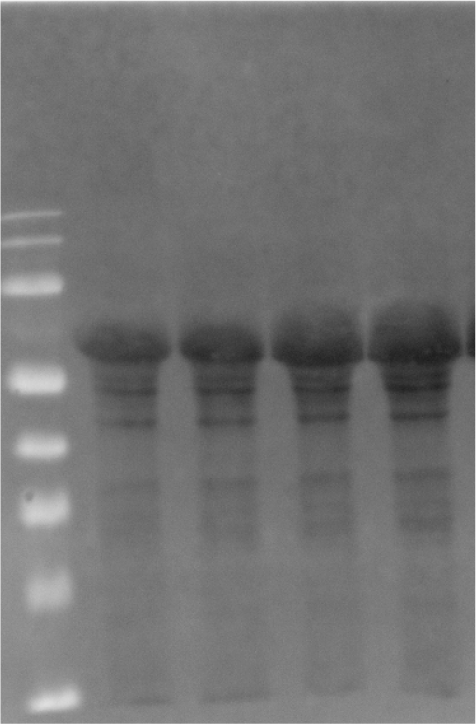

Supplement: Figure 8—source data 2. [file elife-94930-fig8-data2.zip › Figure 8A_Source Data 2/w1_total.tif]

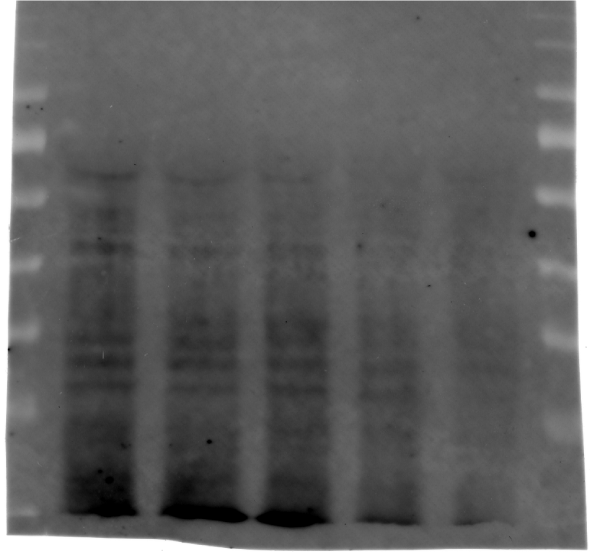

Supplement: Figure 8—source data 2. [file elife-94930-fig8-data2.zip › Figure 8A_Source Data 2/w3_puromycin.tif]

## WEEK1

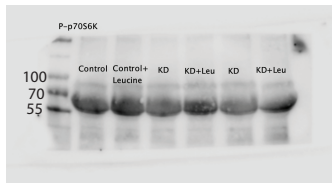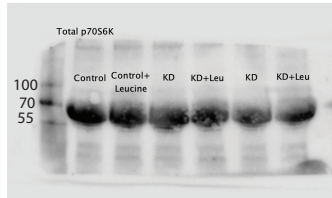

## WEEK2

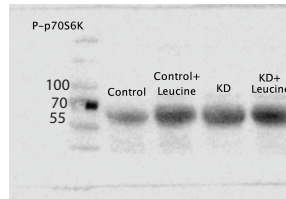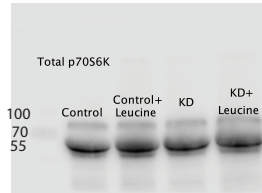

Supplement: Figure 8—figure supplement 1—source data 1. [file elife-94930-fig8-figsupp1-data1.zip › Figure 8-figure supplement 1-source data 1.pdf]

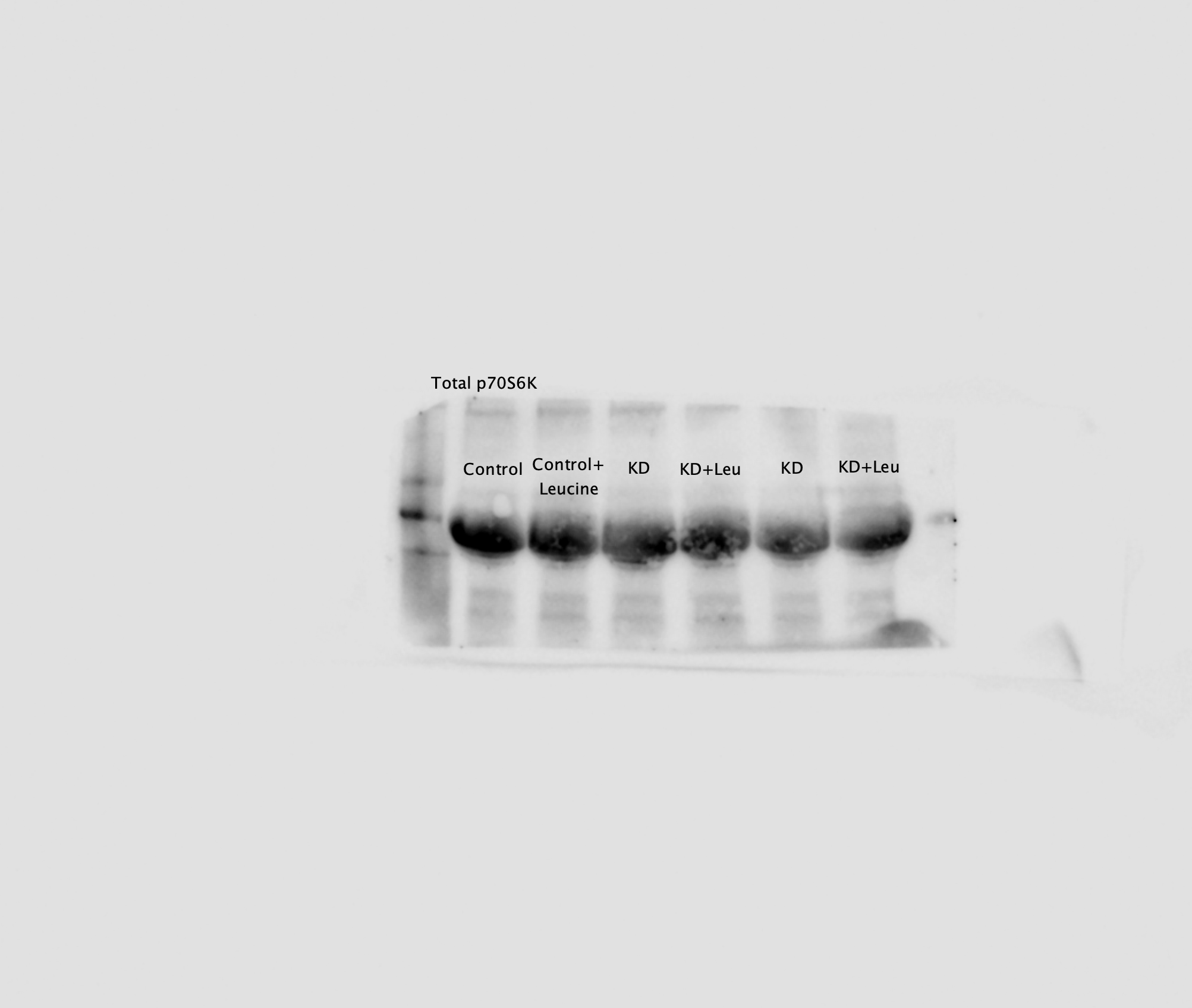

Supplement: Figure 8—figure supplement 1—source data 2. [file elife-94930-fig8-figsupp1-data2.zip › Figure 3-figure supplement 1-source data 2/w1_Total p70S6K.tif]

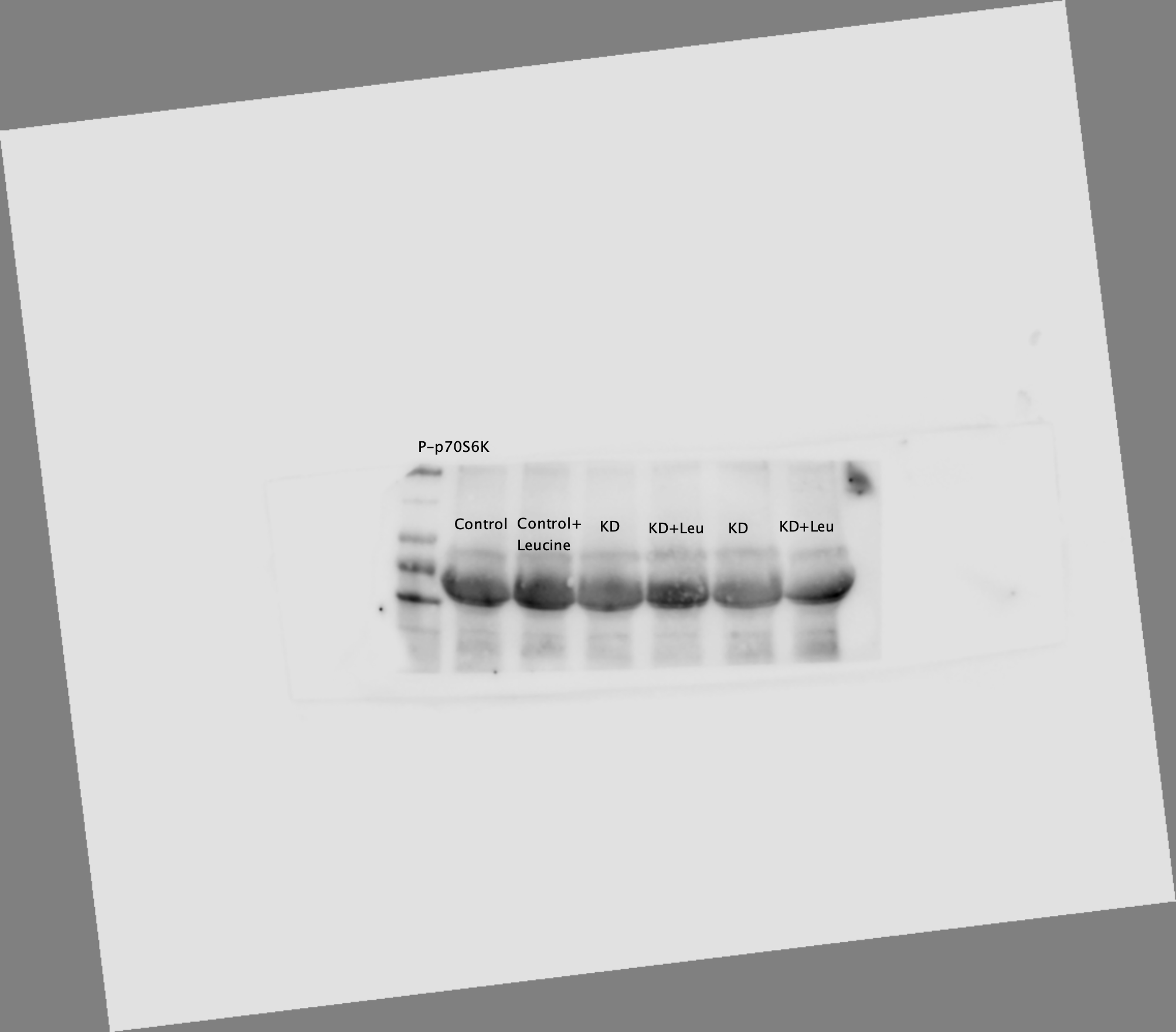

Supplement: Figure 8—figure supplement 1—source data 2. [file elife-94930-fig8-figsupp1-data2.zip › Figure 3-figure supplement 1-source data 2/w1_P-p70S6K.tif]

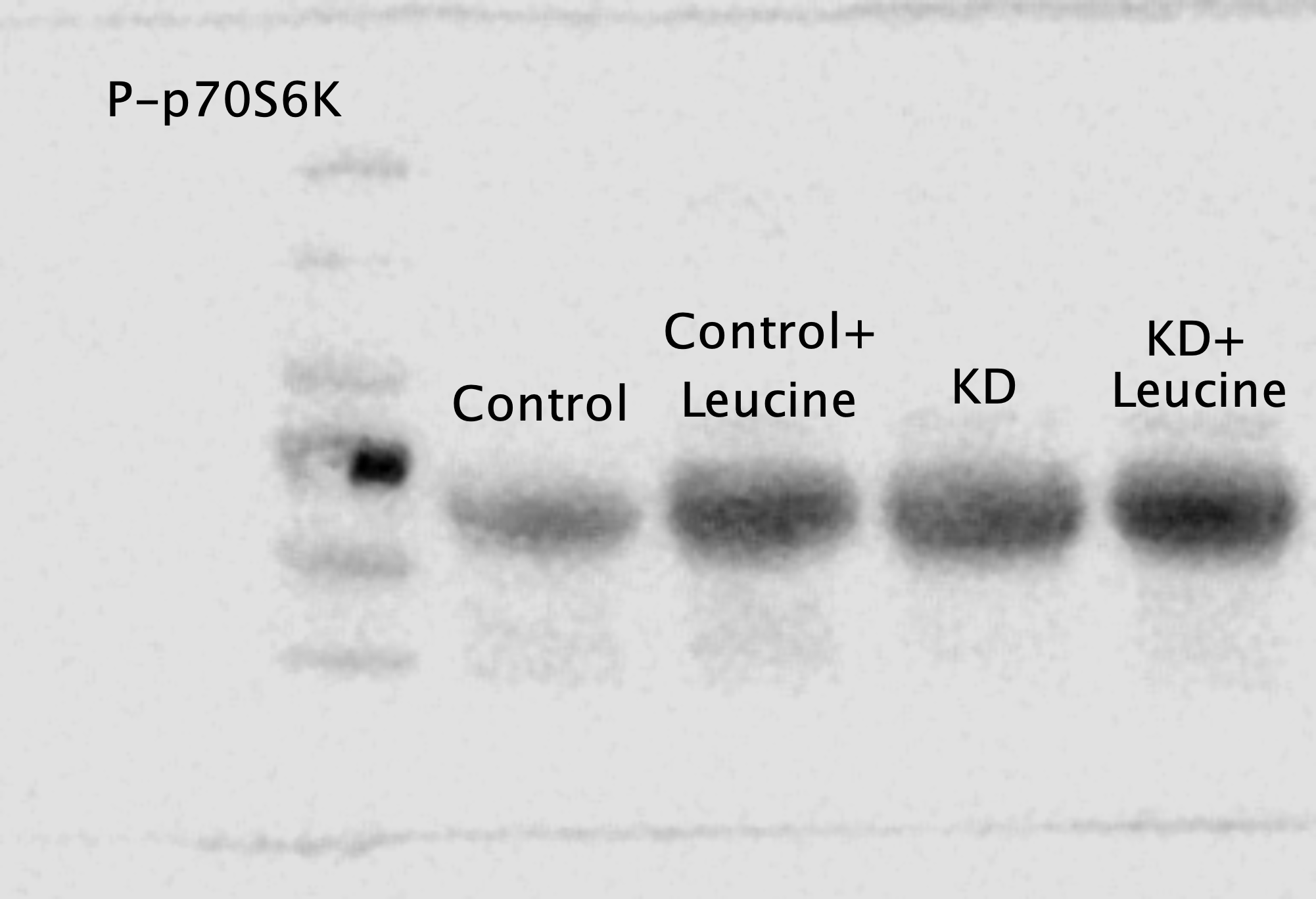

Supplement: Figure 8—figure supplement 1—source data 2. [file elife-94930-fig8-figsupp1-data2.zip › Figure 3-figure supplement 1-source data 2/w2_P-p70S6K.tif]

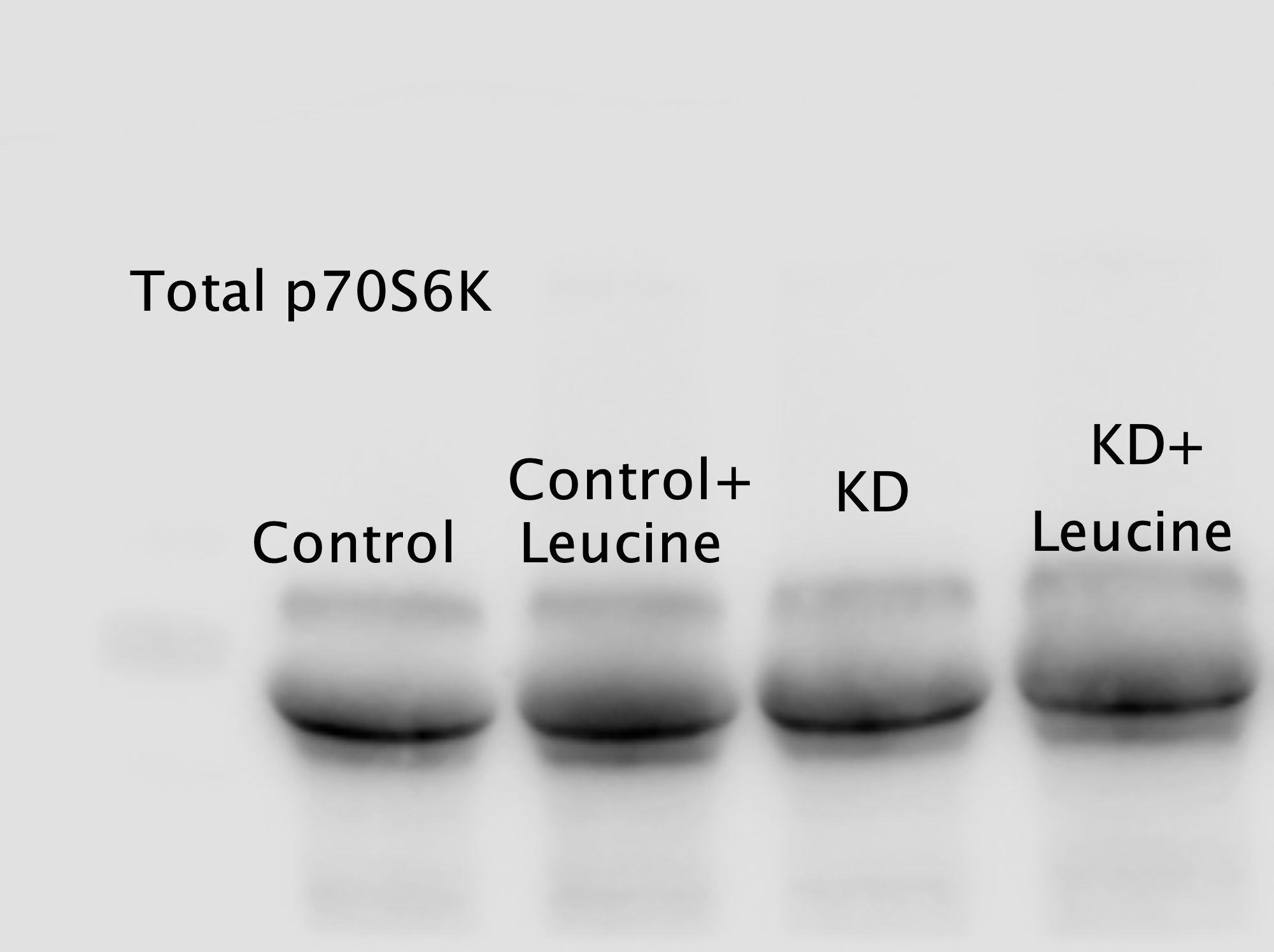

Supplement: Figure 8—figure supplement 1—source data 2. [file elife-94930-fig8-figsupp1-data2.zip › Figure 3-figure supplement 1-source data 2/w2_Total p70S6K.tif]
